# Exogenous naphthaleneacetic acid alleviated alkalinity-induced morpho-physio-biochemical damages in *Cyperus esculentus* L. var. sativus Boeck

**DOI:** 10.3389/fpls.2022.1018787

**Published:** 2022-10-18

**Authors:** Abd Ullah, Fanjiang Zeng, Akash Tariq, Muhammad Ahsan Asghar, Khansa Saleem, Ali Raza, Muhammad Asad Naseer, Zhihao Zhang, Javaria Noor

**Affiliations:** ^1^ State Key Laboratory of Desert and Oasis Ecology, Xinjiang Institute of Ecology and Geography, Chinese Academy of Sciences, Urumqi, China; ^2^ Xinjiang Key Laboratory of Desert Plant Root Ecology and Vegetation Restoration, Xinjiang Institute of Ecology and Geography, Chinese Academy of Sciences, Urumqi, China; ^3^ Cele National Station of Observation and Research for Desert Grassland Ecosystems, Cele, China; ^4^ University of Chinese Academy of Sciences, Beijing, China; ^5^ Department of Biological Resources, Agricultural Institute, Centre for Agricultural Research, ELKH, Martonvásár, Hungary; ^6^ Department of Horticultural Sciences, The Islamia University of Bahawalpur, Bahawalpur, Pakistan; ^7^ Chengdu Institute of Biology, University of Chinese Academy of Sciences, Beijing, China; ^8^ College of Agronomy/Key Laboratory of Crop Physio-ecology and Tillage in Northwestern Loess Plateau, Ministry of Agriculture, Northwest A&F University, Yangling, China; ^9^ Department of Botany, Islamia College University, Peshawar, Pakistan

**Keywords:** salinity, alkalinity, physiology, growth regulators, nitrogen metabolism, antioxidants

## Abstract

*Cyperus esculentus* L. var. sativus Boeck (commonly called Chufa) is a perennial species that produces nutritious underground tubers and contributes to the diet and health of human worldwide. However, it is salt-sensitive and its adaptation to salinity stress remains an enigma. Naphthaleneacetic acid (NAA) plays a vital role in regulating plant salt stress tolerance. Thus, we aimed to investigate the impact of NAA (150 mg/L) application on growth and physio-biochemical response mechanisms of Chufa plants to different levels of salinity stress (0-, 90-, and 180 mM of alkaline stress ([1:1 ratio of Na_2_CO_3_ and NaHCO_3_]). In response to increasing stress levels, shoot-root growth decreased, whereas malondialdehyde (MDA), hydrogen peroxide (H_2_O_2_), osmolytes (soluble protein, proline, and soluble sugars), and activities of superoxide dismutase (SOD), peroxidase (POD), and catalase (CAT) significantly increased. Alkalinity led to significant increase in Na^+^ and Cl^–^, but decrease in Mg^2+^ concentration in both roots and leaves; however, K^+^ decreased significantly in leaves under both stresses. Additionally, ${\text{NO}}_{3}^{-}$ and. levels, nitrate reductase (NR) activities, and glutamate synthase (GOGAT) decreased significantly. However, glutamine synthetase (GS) increased non-significantly at 90 mM but declined at 180 mM. Foliar NAA application reduced Na^+^ and Cl^-^, MDA, and H_2_O_2_ but increased photosynthetic pigments, K^+^ and Mg^2+^, osmolytes, nitrogen (N) metabolism, and upregulating the enzymatic antioxidant system to reduce oxidative stress under alkaline conditions. Hence, our findings manifest that NAA application is an effective strategy that can be utilized to enhance tolerance of chufa plants to alkaline stress. Future studies should explore whether NAA can positively alter the nutrient composition of chufa tubers at deeper molecular levels, which might offer solutions to nutritious problems in developing countries.

## Introduction

Salinization and alkalization of soils have become major socio-economic issues that have negative impacts on crop growth and productivity (Li et al., 2014; Hassani et al., 2021). In general, soil alkalinity occurs simultaneously with soil salinity. It is estimated that more than 25% of the earth’s surface is covered by alkaline soils, affecting more than 434 million hectares of land (FAO, http://www.fao.org/soils-portal/soil-management). Alkalinity is caused by carbonates and bicarbonates, which are common constituents of irrigation water. They adversely affect physiological homeostasis (Munns and Tester, 2008; Guo et al., 2019) by altering the ionic balance within plant cells (Li et al., 2017). Alkalinity, for example, causes the high pH in the rhizosphere, which reduces the availability and uptake of important nutrients such as K^+^, Ca^2+^, Mg^2+^, ${\text{NO}}_{3}^{-}$ , and ${\text{H}}_{2}{\text{PO}}_{4}^{-}$ , by causing their precipitation (Yang et al., 2007; Ullah et al., 2019). Plants can respond to alkaline salt stress by modifying certain metabolic processes, such as ion transport and accumulation, photosynthesis, osmotic solute accumulation, and hormone synthesis, and nitrogen metabolism (Shi and Wang, 2005; Yang et al., 2009; Wang et al., 2012; Ullah et al., 2019). Alkalinity stress also causes an increase in antioxidant processes, which is one of the mechanisms for the scavenging of free radicals under stress (Mir et al., 2018). Nitrogen (N) metabolism is of great importance to plants, as it provides proteins and nucleic acids that control many of their cellular functions. Plants absorb nitrogen from the soil primarily as nitrates ${\text{(NO}}_{3}^{-})$ and ammonia ${\text{(NH}}_{4}^{+})$ (Luo et al., 2013). Next, ${\text{NO}}_{3}^{-}$ is reduced into nitrite ${\text{(NO}}_{2}^{-})$ and then into ${\text{NH}}_{4}^{+}$ through the actions of nitrate reductase (NR) and nitrite reductase (NiR) enzymes, respectively. In addition, ${\text{NH}}_{4}^{+}$ is assimilated into amino acids through glutamine synthetase (GS) and glutamate synthase (GOGAT) cycle or through glutamate dehydrogenase (GDH) (Shi et al., 2009; Ullah et al., 2019). N is an essential constituent of amino acids, proteins, amides, and polyamines and secondary metabolites and hence interferes in several physio-biochemical processes. Its regulations therefore, contribute to salinity stress tolerance in plants (Zaki, 2016; Arghavani et al., 2017). However, salinity stress and N metabolism interact in a complex manner affecting nearly almost every physiological process in plants (Läuchli and Lüttge, 2002). However, previous studies have demonstrated that alkaline stress has a far greater impact on nitrogen metabolism than saline stress. Several studies have shown that alkaline stress impacts nitrate assimilation and/or uptake, resulting in lower concentrations of nitrate in Suaeda glauca (Yang et al., 2008), barley (Yang et al., 2009), blackseed grass (Yang et al., 2010), and soybeans (Ullah et al., 2019). Consequently, regulating N metabolism might be as important for alkaline-tolerance as for salt-tolerance.

Normal growth conditions produce relatively low levels of reactive oxygen species (ROS) such as hydrogen peroxide (H_2_O_2_) and superoxide anion radical $\left(O_{2}^{-}\right)$ . However, saline-alklaine stress conditions disrupt this homeostasis, and causes excessive ROS production, leading to intracellular oxidative stress. In response plants activates enzymatic [(superoxide dismutase (SOD), catalase (CAT) and peroxidase (POD)] and non-enzymatic antioxidant defence mechanisms to eliminate the excess ROS and protect plants cells from oxidative damage (Guo et al., 2019; Sarker and Oba, 2020).

Moreover, several studies have demonstrated that alkaline stress has a more severe effect on plants than saline stress (Yang et al., 2009; Wang et al., 2012; Ullah et al., 2019). For instance, saline stress generally causes ionic damage and osmotic stress in plants (Kiełkowska et al., 2019), whereas alkaline stress leads high pH injuries along with the above-mentioned damages (Yang et al., 2009; Mao et al., 2019). The studies comparing the effects of alkaline stress on *Lathyrus quinquenervius* and *Glycine soja* revealed that the former is more damaging than the latter (Zhang & Mu, 2009; Ullah et al., 2019). In the case of alkalinity, for example, there was a greater reduction in the growth, photosynthesis, ionic regulation, carbon and nitrogen metabolism of soybean (Ullah et al., 2019). Likewise, alkaline stress inhibited germination, root system activities, photosynthesis, organic acid imbalance, reactive oxygen species (ROS), and malondialdehyde (MDA) concentrations, resulting in impaired growth (Zhang and Mu, 2009). However, there have been few studies examining the effects of alkaline stress in plants and the mechanisms by which plants adapt to alkaline stress, as compared to saline stress (Ahmad et al., 2014; Hu et al., 2015). It is therefore imperative to unravel the physio-biochemical mechanisms by which crop species, respond to alkaline stress in order to meet the growing population’s food needs in the face of climatic changes and diminishing fresh water resources.

Phytohormones, also known as plant growth regulators, are molecules derived from the plant biosynthetic pathway that can mediate the growth and development of plants both under normal and stressed conditions. Several phytohormones, including auxins (IAA), gibberellins (GA), cytokinins (CKs), brassinosteroids (BRs), and ethylene (ETHY), regulate plant growth and development in a coordinated fashion. These phytohormones influence a variety of physiological and biochemical processes (Fahad et al., 2015). They enhance abiotic stress tolerance and productivity of economic crop species (Iqbal et al., 2012; Fahad et al., 2015). Foliar applications of growth regulators are recommended as considerably quick and timely approach to achieve tolerance in plants grown in salinity-alkalinity-affected soils (Mir et al., 2018; Nigam et al., 2022). Naphthalene acetic acid (NAA) is a synthetic plant hormone, similar to naturally occurring indole acetic acid (IAA), in the auxin family. It stimulates cell division, elongation, membrane permeability, leaf chlorophyll content, photosynthesis, mRNA synthesis, water uptake and other physiological processes (Basuchaudhuri, 2016; Ullah and Sajjad, 2017). Plant growth regulators, whether natural or synthetic, affect endogenous hormonal patterns in the plant, either by supplementing sub-optimal levels or by interfering with their synthesis, translocation, or inactivation (Basuchaudhuri, 2016). Foliar application of plant growth regulators, including NAA, have been reported to increase, plant photosynthesis, ions regulation and anti-oxidant defense mechanism and hence protect plant tissues from salinity-induced damages (Ullah and Sajjad, 2017; Muhammed et al., 2022). However, our understanding of the role of exogenous NAA application in alleviating the alkalinity-induced physio-biochemical responses of plants is sparse, and needs further investigation.

The cultivated yellow nutsedge (*Cyperus esculentus* L. var. sativus Boeck), commonly known as chufa, is a perennial crop plant (Cyperaceae family). It produces underground almond-like tubers that are remarkably sweet and nutritious, having several benefits to human health (Pascual et al., 2000; Sánchez‐Zapata et al., 2012). Its tubers are also used in preparing a non-alcoholic, milk-like drink, known as horchata, which has been the subject of recent studies (Sebastia et al., 2010; Sánchez‐Zapata et al., 2012). Additionally, this crop has aphrodisiac, carminative, diuretic, stimulant, emmenagogue, and tonic properties and is commonly used to treat excessive stomach gassiness, indigestion, diarrhea, and dysentery (Adejuyitan, 2011). The tubers of chufa contain many nutrients and bioactive compounds, making them an extremely important cash crop for humans and animals (Adejuyitan, 2011; Sánchez‐Zapata et al., 2012; Maduka and Ire, 2018). Despite of being neglected, it has become an increasingly important crop due to the health benefits and nutritional value they provide. Chufa plant has many uses in the food industry, for instance, its flour is now commonly used to thicken bread, cakes, or prepared into alcoholic and nonalcoholic beverages. In addition to providing dietary diversification to alleviate micronutrient deficiency, especially amongst the poor and children, chufa can contribute to the agricultural gross domestic product (GDP) both locally and internationally (Asare et al., 2020). Given the rising human population, this neglected and underutilized species can contribute to food security and poverty reduction. This species prefers moist, sandy soils and are tolerant of drought and flooding as well as temperature fluctuations in the soil, but not tolerant of salinity (Halverson, W. L. 2003). A variety of aspects related to cultivating chufa have been thoroughly examined, including, cultivar selection and plant characterization, crop management techniques, irrigation, nutrition and fertilization (Pascual-Seva et al., 2018). However, to the best of our knowledge, investigation of salinity-alkalinity responsive mechanism is entirely missing in the case of salt sensitive Chufa plant species. Therefore, examining its growth and physio-biochemical changes in response to soil alkalinity is imperative.

This study aimed to investigate, how alkalinity impact growth Chufa plant in terms of physiological and biochemical changes. We hypothesized that, alkalinity stress (high pH) would decrease the growth of chufa plants by disrupting their metabolism; however, exogenous NAA application might enhance their growth by alleviating the negative effects associated with alkalinity stress. To test our hypothesis, we have performed morphological and physio-biochemical investigations to gain deeper insights into the salinity tolerance mechanism of chufa plants by evaluating various parameters, including, (i) Shoot and root growth and biomass, (ii) photosynthetic chlorophyll pigments, (iii) salt ions accumulation, (iv) Lipid peroxidation and reactive oxygen species, (v) enzymatic antioxidant mechanism (vi) osmolytes accumulation and (vii) nitrate $\left(NO_{3}^{-}\right)$ reduction and ammonium $\left(NH_{4}^{+}\right)$ assimilation in response to increasing levels of simulated alkalinity stress conditions and foliar NAA application.

## Materials and methods

### Study area and growth conditions

We conducted this study at the Cele National Station of Observation and Research for Desert-Grassland Ecosystem (37° C00′ 56′′ N, 80° C43′ 81′′ E), Chinese Academy of Sciences. It is located at the southern fringe of a hyper-arid saline desert known as the Taklamakan. The mean yearly temperatures, precipitation, and evaporative potential are 11.9°C, 35 mm, and 2600 nm, respectively. We obtained the tubers of *Cyperus esculentus* L. var. sativus Boeck (Fengchan No1) chufa plant from Xinjiang Institute of Ecology and Geography, Chinese Academy of Sciences, Urumqi, China. In August 2022, four same-sized healthy tubers were sown in each 1.5 L plastic pot (Height, 12.0 cm; top diameter, 15.01 cm; and basal diameter 11.02) with a 2 cm hole at the bottom (ca. 18%), filled with 1 kg of homogenized soil (aeolian loamy sand with organic C, 2.99 g kg^−1^; total N, 0.23 g kg^−1^; total P, 0.60 g kg^−1^ and total K, 23.11 g kg^−1^, pH 8.43, and EC 177.7 μs.cm^-1^) The pots were arranged in a complete randomized block design (RCBD) in August 2022. During the first three weeks, water was supplied every three days to each pot using a weight method to field capacity (18% w/w). In the study area, groundwater has a salinity of approximately 40-50 mM Na^+^ and is ordinarily used for local irrigation.

### Treatments and experimental design

At the fifth leaf stage (four weeks after sowing), we selected 60 pots (2 seedlings/pot) with uniform seedlings and divided them into six groups for the alkaline stress (1:1 ratio of Na_2_CO_3_ and NaHCO_3_) and NAA application. Three groups for alkaline stress treatments were treated with (i) 0 mM (controlled condition), (ii) 90 mM, and (iii) 180 mM alkaline stress. The other three groups were subjected to the same three levels of alkaline stress but applied with NAA application (150 mg/L) using a sprinkler four times (20, 30, 35, and 40 days after sowing). In a preliminary growth experiment, NAA concentrations ranging from 50 to 150 mg were studied (**Table S1**). We selected the optimal concentration of NAA based on improved growth of chufa seedlings under 90 mM alkalinity stress. Each treatment was replicated three times. Finally, we harvested the 50-days old plants, and were immediately frozen in liquid nitrogen and stored (-80°C) for the determination of physiological indexes.

### Measurement of plant growth and biomass

After harvesting, the shoot height and root length were measured using a measuring tape. Next fresh and dry weights, and shoot and root dry weights of the seedlings were measured using an electric balance. For dry weight determination, the plants were oven-dried at 105° C for 30 min and then dried at 75° C until constant weight (Shao et al., 2015).

### Measurement of photosynthetic pigments

The chlorophyll (0.1–0.3 g fresh leaves) was extracted from the leaves using ethanol (95 percent, vol/vol) and measured at 665 nm and 649 nm following a standard method (Lichtenthaler and Buschmann, 2001) calculated the chlorophyll contents using the following equations (mg g^-1^ FW).


(1) Chl a = 13.98 A665 - 6.88 A649

(2) Chl b = 24.96 A649 - 7.32 A665

(3) Chl a/b = Chl a / Chl b

(4) Chl = Chl a + Chl b

### Determination of mineral elements

Dry leaf and root samples oven dried at 105° C for 30 min and then grinded into fine powder using an electric mortar. Following this, the finely powdered samples (0.05 g) were transferred into a centrifuge tube with deionized water (4 ml) and placed in a boiling water bath for 40 min. After centrifuging the tubes for 10 min at 4000 rpm, the supernatant was collected. The concentrations of sodium (Na^+^), potassium (K^+^), and (magnesium) (Mg^2+^) ions were determined using an atomic absorption spectrophotometer (Super 990F, Beijing Purkinje General Instrument Co. Ltd. Beijing, China) (Mostofa et al., 2015). Furthermore, Cl^−^ ion concentration was determined using ion chromatography (DX-300 ion chromatographic system, AS4A-SC chromatographic column, CDM-II electrical conductivity detector, mobile phase: Na_2_CO_3_/NaHCO_3_ = 1.7/1.8 mM; Dionex, Sunnyvale, CA, USA) (Ullah et al., 2019).

### Determination of H_2_O_2_ and cell membrane injury

Hydrogen peroxide (H_2_O_2_) concentrations were determined by a standard procedure (Patterson et al., 1984). The 0.2 g freshly leaf samples were homogenized in 5 ml of trichloroacetic acid (TCA) (0.1%) in an ice bath, transferred to tubes, and centrifuged at 5000 × *g* for 10 min (4°C). Next, we centrifuged the supernatant comprising 0.1 ml of titanium reagent (50 µL of 20% titanium tetrachloride) and 0.2 ml of ammonia, centrifuged at 10,000 × *g* for 10 min. Following five washes with acetone, the precipitate was centrifuged at 10,000 × *g* for 10 min, after which 3 ml of 1 M H_2_SO_4_ was added.

Malondialdehyde (MDA) concentration was assessed based on the thiobarbituric acid (TBA) test for the evaluation of lipid peroxidation (Heath and Packer, 1965). Fresh leaf samples (0.5 g) were homogenized in 1ml of 5% TCA and centrifuged for 10 min at 5,000 × *g* (4°C). Using a separate tube, 4 ml of the supernatant was added to 2 ml of 20% TCA, and the mixture was heated at 100°C for 15 min before centrifugation at 5,000 × *g* for 10 min. A spectrophotometer was used to measure absorbance at 450, 532, and 600 nm, and the concentration of MDA was calculated according to the following equation:


$${\text{MDA (mol g}}^{-1}FW) = = 6\text{.45 (A532 - A600) - 0}.56A450$$

### Antioxidant enzyme activities

Fresh leaves samples were ground and homogenized in a chilled mortar with 0.1 M phosphate buffer (pH 7.3) and 0.5 mM ethylenediaminetetraacetic acid (EDTA). The homogenate was centrifuged at 8000 × *g* (10 min at 4°C). The activity of SOD was assayed by measuring the reduction rate of nitroblue tetrazolium (NBT) at 560 nm (Giannopolitis and Ries, 1977). One unit of SOD activity was defined as the amount of enzyme required for 50% inhibition of NBT reduction at 560 nm. Moreover, with minor modifications, the POD activity was determined according to standard methods (Wang et al., 2018), with minor changes. A reaction mixture was prepared by mixing 2 ml of buffer substrate (8 mM guaiacol and 100 mM Na_3_PO_4_ pH 6.4), 24 mM H_2_O_2_ in 0.5 ml of enzyme extract). At 460 nm, absorbance values were measured twice at 1-minute intervals.

We calculated enzyme activity by increasing the absorbance of the reaction system by 0.01 up to a maximum of 1U per min, which was then converted into U/g·min^−1^. CAT activity was determined by monitoring the disappearance of H_2_O_2_ (Sabra et al., 2012). Initially, 50 ml of enzyme extract was poured into 1.5 ml of reaction mixture containing 50 mM K-phosphate buffer (pH 7.0) and 15 mM H_2_O_2_. One unit of CAT corresponds to one mole of H_2_O_2_ degradation per minute measured at 240 nm for 1 min. The absorbance was recorded at 240 nm for 1 min. One unit of CAT corresponds to one mole of H_2_O_2_ degradation per min.

### Determination of N-metabolizing enzymes

The nitrate reductase activity was determined by homogenizing 0.2 g using 2 ml of 25 mM phosphate buffer saline (PBS, pH 8.7), which contained 10 mM cysteine and 1 mM EDTA, and then centrifuging for 20 min at 30,000 × *g*. the resulting supernatant was tested for NR activity using a diazo-coupling method employing Griess reagent (Sánchez et al., 2011).

The GS activity was determined by homogenizing them in 2 ml of 50 mM TrisHCl buffer (pH 7.8; containing 15% glycerol, 0.1% TritonX-100, 1 mM of EDTA, and 14 mM of 2-mercaptoethanol) and centrifuging them twice at 4°C for 10 min. After complexing with acidified ferric chloride, the supernatant was used to determine GS by forming glutamine hydroxamate using a 540 nm fluorescence measurement (Liu et al., 2014).

### Determination of soluble sugar, proline and soluble protein

We ground dried leaves samples into a fine powder using a ball mill. Next, the powdered samples were added to a centrifuge tube containing 2 ml of 80% ethanol. Following incubation at 80°C in a shaking water bath (30 min), the mixture was centrifuged at 4,000 g for 5 min. Further two extractions were performed using 80% ethanol on the pellets. We retained, combined and stored the supernatant at 20°C for soluble sugar determination, following a standard method (Yemm and Willis, 1954). Proline extraction (0.2 g samples) was conducted using two ml of 10% acetic acid and five ml of 3% salicylic acid, respectively. The mixture was centrifuged at 12,000 × *g* for 10 min. The resulting supernatants were analyzed using a standard method (Liu et al., 2014). For soluble protein determination, extracts were made from 0.3 g of frozen fresh leaf samples in sodium phosphate buffer (50 mmol, pH 7.8), and centrifuged at 4000 × *g* for 10 min (4°C). The concentration of soluble proteins using bovine serum albumin as the standard (Bradford, 1976).

### Statistical analysis

Measurements were replicated three times and sorted using Microsoft Excel 2019. SPSS version 16.0 (Chicago, IL, United States) was used to perform descriptive statistics and one-way analysis of variance (ANOVA). Duncan’s multiple range tests were used to compare means at a significance level of 0.05. GraphPad Prism 8 was used to create the figure graphics. For interpretation purposes, Pearson correlation analyses were conducted using OriginPro 2019 software (Origin Lab Carporation Northampton, MA, USA) regarding the growth parameters, chlorophyll pigment concentrations in the leaf samples, N metabolism, osmolytes accumulation, mineral nutrition, and reactive oxygen species production rate, as well as antioxidant enzymatic activities.

## Results

### NAA-induced alkaline stress alleviation in chufa growth parameters

**Figure 1:**
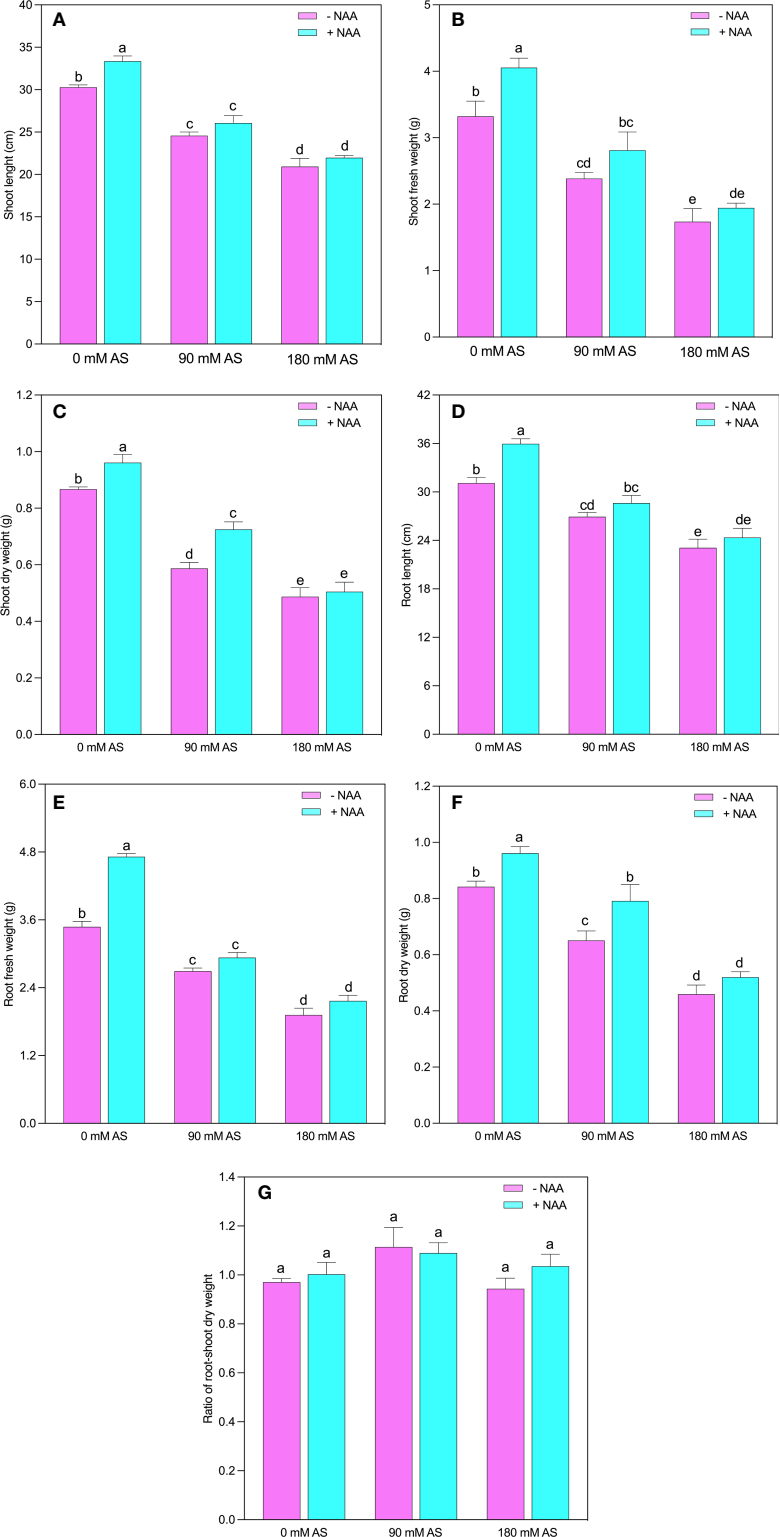
Changes in **(A)** shoot length, **(B)** shoot fresh weight and **(C)** shoot dry weight, **(D)** root length, **(E)** root fresh weight **(F)** root dry weight and, **(G)** root-shoot ratio of chufa plants under alkaline stress (AS) and exogenous naphthaleneacetic acid (NAA) application. Bars represents SD of mean, n=3. Different letters indicate significantly different values at *P*<0.05 (Duncan’s method).

Both levels of alkaline stress produced a substantial reduction in growth parameters of chufa plant (**Figures 1A-G**). The SL, SFW, SDW, RL, RFW, and RDW experienced the 18.8, 28.2, 32.2, 13.0, 22.6, and 22.8% reduction in 90 mM alkaline stress and 30.9, 47.8, 43.8, 25.7, 44.8, and 45.4% inhibition after 180 mM alkaline stress, respectively (Fig 1). While, the exogenous application of NAA improved the SL, SFW, SDW, RL, RFW, and RDW 10, 22, 11.0, 15.4, 35.4, and 14% relative to untreated plants, 5.8, 18.0, 23.5, 6.2, 8.8, and 21.5% as compared to 90 mM alkaline stress, and 5.0, 12.0, 3.6, 5.3, 12.6, and 13.0% after 90 mM alkaline stress, respectively. The root-shoot ratio exhibited the inverse trend of other growth indices since it did not display any substantial alterations after either treatment relative to control plants (**Figure 1**).

### Ions regulation in chufa plants under alkaline stress followed by NAA supplementation

**Figure 2:**
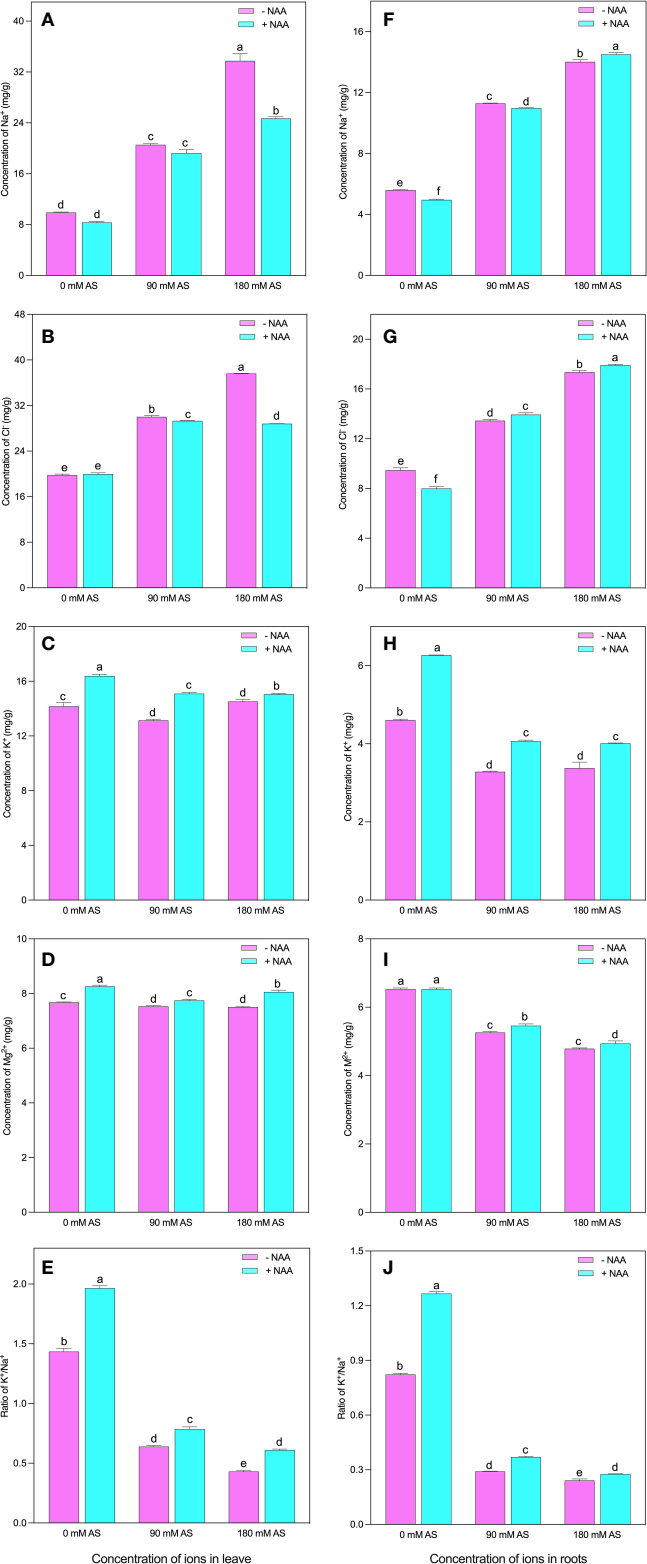
Changes in ions concentration of leaf **(A)** Na^+^, **(B)** Cl^-^ **(C)** K^+^ **(D)** Mg^++^ and **(E)** K^+/^Na^+^ ratio and root **(F)** Na^+^, **(G)** Cl^-^ **(H)** K^+^ **(I)** Mg^++^ and **(J)** K^+/^Na^+^ ratio of chufa plants under alkaline stress (AS) and exogenous naphthaleneacetic acid (NAA)application. Bars represents SD of mean, n=3. Different letters indicate significantly different values at *P*<0.05 (Duncan’s method).

In both the studied organs (leaves and roots), the plants showed abrupt modifications in the nutritional profile under alkaline stress followed by NAA application (Fig 2A-J). Particularly, in leaves of chufa plant, the concentration of Na^+^ and Cl^-^ were greatly elevated under both alkaline stress levels. For instance, a 3.4-fold enhancement was noted in Na^+^ concentration, and 1.5- and 1.9-fold up-regulation in Cl concentration was experienced by the chufa leaves when exposed to 90 mM and 180 mM alkaline stress, respectively (**Figures 2A, B**). Nevertheless, the NAA exogenous application declines the concentrations of Na^+^ by 15.6, 6.5, and 27.0% under 0 mM, 90 mM, and 180 mM alkaline stress, respectively. Interestingly, the highest reduction (23.7%) for Cl- concentration was seen when the plants were grown under 180 mM alkaline stress (**Figures 2A, B**).

Contrarily, the K^+^, Mg^2+,^ and K^+^/Na^+^ concentrations were observed to be declined in the alkaline stress-affected plants except in the case of K^+^ under higher alkaline levels (**Figures 2C-E**). In contrast, the 90 mM alkaline stress-induced 7.5% reduction in K^+^ concentration of chufa. leaves between Mg^2+^ and K^+^/Na^+^, the most pronounced inhibition (55.3 and 70%) was detected in K^+^/Na^+^, when the plants were cultivated in 90 mM and 180 mM alkaline stress, respectively. Nonetheless, the NAA exogenous application induced the improvement of 15.0, 15.0, and 3.6% in K^+^, 7.6, 3.0, and 7.3% in Mg^2+^, and 37.0, 22.6, and 41.5% in K^+^/Na^+^ in the 0 mM, 90 mM, and 180 mM alkaline stress-treated plants, respectively (**Figures 2C-E**).

All the nutrients exhibited similar trends in the case of roots (**Figures 2F-J**). The 90 mM and 180 mM levels of alkaline stress produced the upregulation of Na^+^ by 2.0- and 2.5-fold, and Cl^-^ by 1.4- and 1.8-fold, respectively **Figures 2F, G**). Further, the NAA supplementation enhanced the Na^+^ and Cl^-^ concentrations in all the treatments except Cl^-^ concentration at 0 mM alkaline stress. However, the alkaline stress (90 mM and 180 mM) profoundly reduced the K^+^ concentration by 28.7 and 26.6%, the Mg^2+^ concentration by 19.4 and 26.6%, and the K^+^/Na^+^ concentration by 64.7 and 70.6%, respectively (**Figures 2H-J**). Contrarily, the NAA application significantly improved the K^+^, Mg^2+^, and K^+^/Na^+^ concentrations except for Mg^2+^, where the plants were not treated with alkaline stress (**Figures 2H-J**).

### Modifications in chlorophyll pigments under alkaline stress followed by NAA application

**Figure 3:**
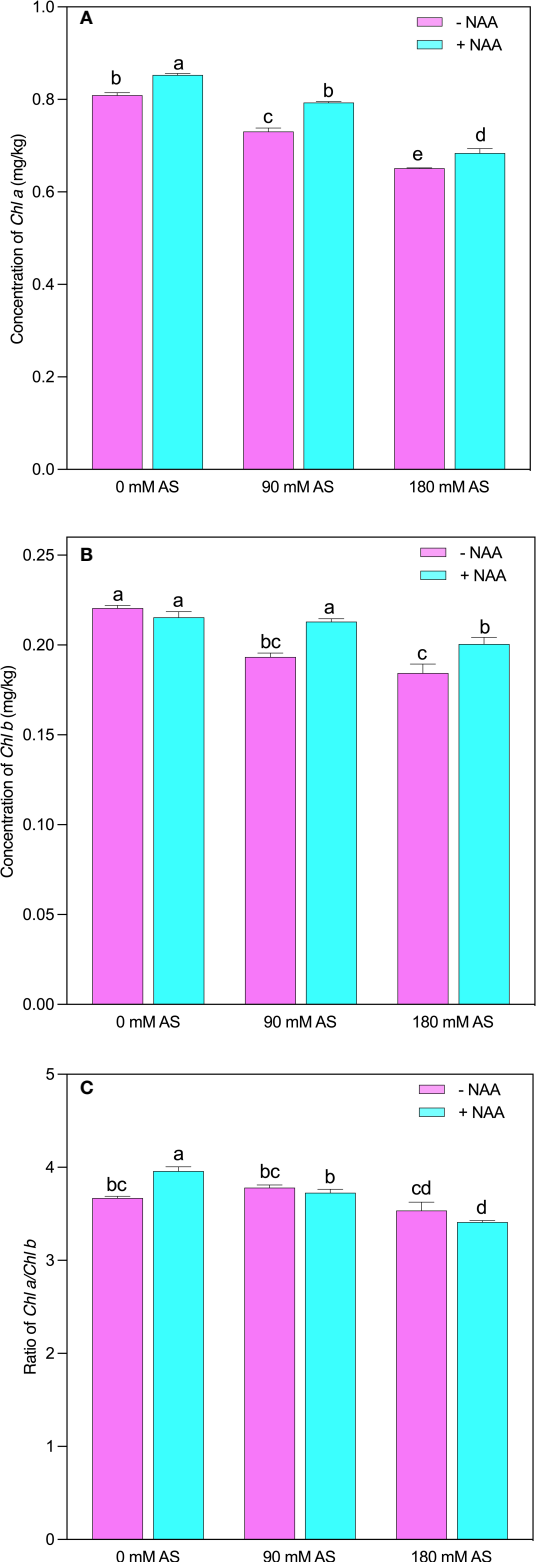
Changes in concentrations of **(A)** chlorophyll *a* **(B)** chlorophyll *b* and **(C)** ratio of chlorophyll *a*/*b* in chufa plants under alkaline stress (AS) and exogenous naphthaleneacetic acid (NAA) application. Bars represents SD of mean, n=3. Different letters indicate significantly different values at *P*<0.05 (Duncan’s method).

The chufa plants showed lower chlorophyll contents after alkaline stress compared to untreated plants (**Figures 3A-C**). This inhibition was also seen in the case of NAA application. Briefly, the chl-*a* and chl–*b* showed a 9.6 and 12% reduction after 90 mM alkaline stress, while 180 mM alkaline stress-induced 19.5 and 16% decline in Chl-*a* and –*b* contents, respectively. However, a significant improvement was shown by the chufa plants when the NAA was applied under both stress levels (**Figures 3A, B**). The Chl a/b presented an interesting trend as it was not remarkably modified after both stress levels. In addition, the plants displayed only significant change when treated with NAA supplementation under no alkaline stress (**Figure 3C**).

### Reduction of MDA and H_2_O_2_ levels by NAA application under alkaline stress

**Figure 4:**
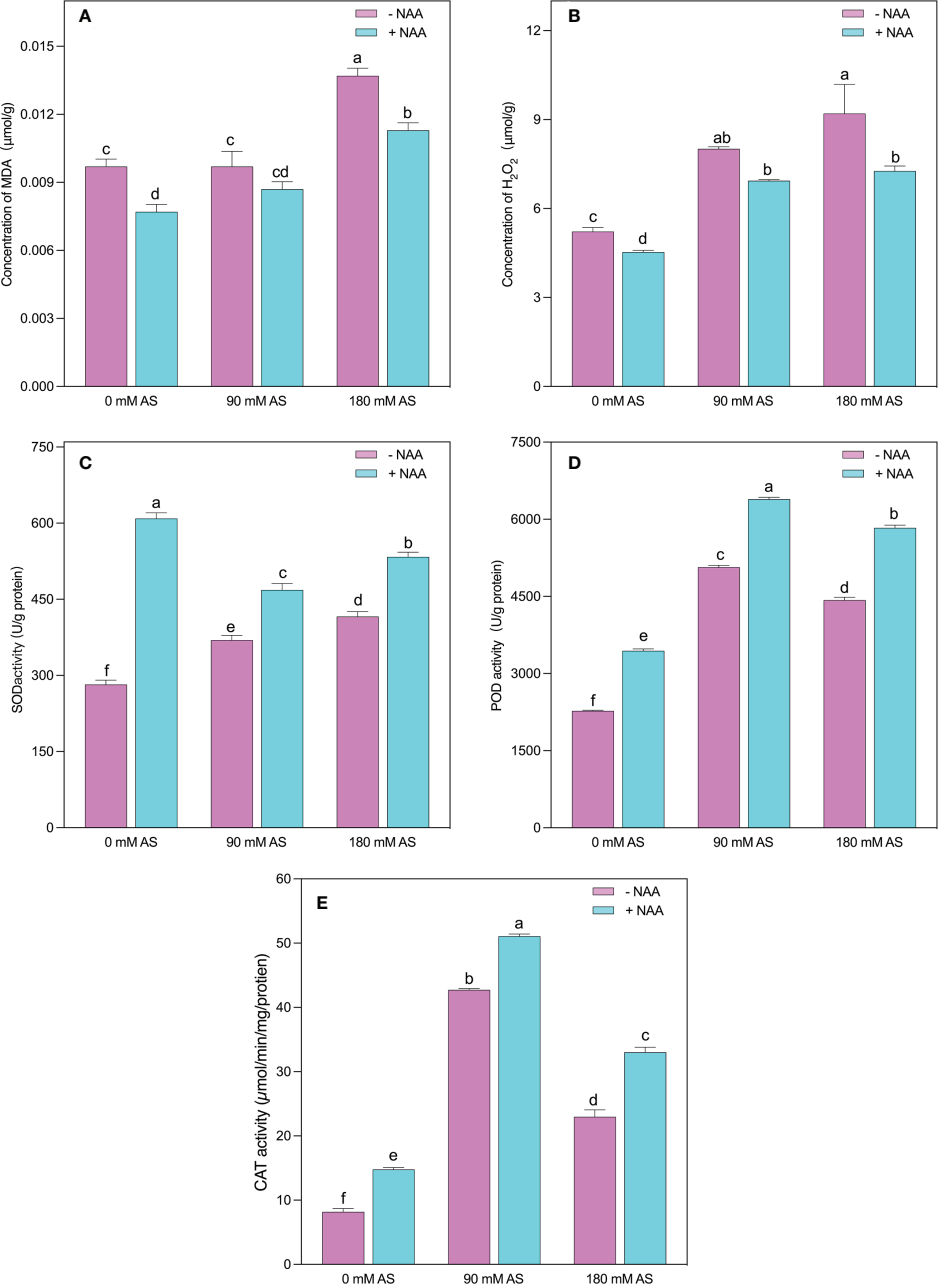
Changes in concentrations of **(A)** MDA **(B)** H_2_O_2_ and enzymatic activity of **(C)** SOD **(D)** POD, and **(E)** CAT in chufa plants under alkaline stress (AS) and exogenous naphthaleneacetic acid (NAA)application. Bars represents SD of mean, n=3. Different letters indicate significantly different values at *P*<0.05 (Duncan’s method).

The higher accumulation of MDA and H_2_O_2_ indicates oxidative damage in plants (**Figures 4A, B**). The same was the case in our experiment since the alkaline stress-subjected plants showed abrupt elevation in MDA and H_2_O_2_ levels. Interestingly, after 90 mM alkaline stress, the chufa. plants did not show substantial up-regulation in MDA levels. However, a 30% increase was recorded in the 180 mM alkaline stress-treated plants. Nevertheless, the NAA-treated plants displayed a 20, 10, and 15.4% decrease in MDA levels after 0 mM, 90 mM, and 180 mM alkaline stress, respectively (**Figures 4A, B**).

In contrast to MDA, the H_2_O_2_ levels were remarkably up-regulated (53.3 and 76%) by both respective alkaline concentrations. Nonetheless, the NAA application resulted in a 13, 13.5, and 21% decline in H_2_O_2_ levels when applied under 0 mM, 90 mM, and 180 mM alkaline stress conditions, respectively (**Figures 6A, B**).

### Enhancement in antioxidant enzymes under alkaline stress followed by NAA supplementation

The plants activate their antioxidant machinery to deal with higher production of MDA and H_2_O_2_, as evident in SOD, POD, and CAT activities (**Figures 4C-E**). More specifically, a 1.3-, 5.2-, and 2.2-fold increment was detected in SOD, POD, and CAT activities when the chufa. plants were subjected to 90 mM alkaline stress, respectively. Moreover, the 180 mM alkaline stress induced the elevation of 1.5-, 2.8-, and 2-fold in SOD, POD, and CAT activities, respectively. The NAA application significantly improved the SOD activities by 2.1-, 1.2-, and 1.3-fold, POD activities by 1.8-, 1.2-, and 1.4-fold, and CAT activities by 1.5-, 1.3-, 1.3-fold under 0 mM, 90 mM and 180 mM alkaline stress, respectively (**Figures 4C-E**).

### Alterations in N-metabolism under alkaline stress followed by NAA addition

**Figure 5:**
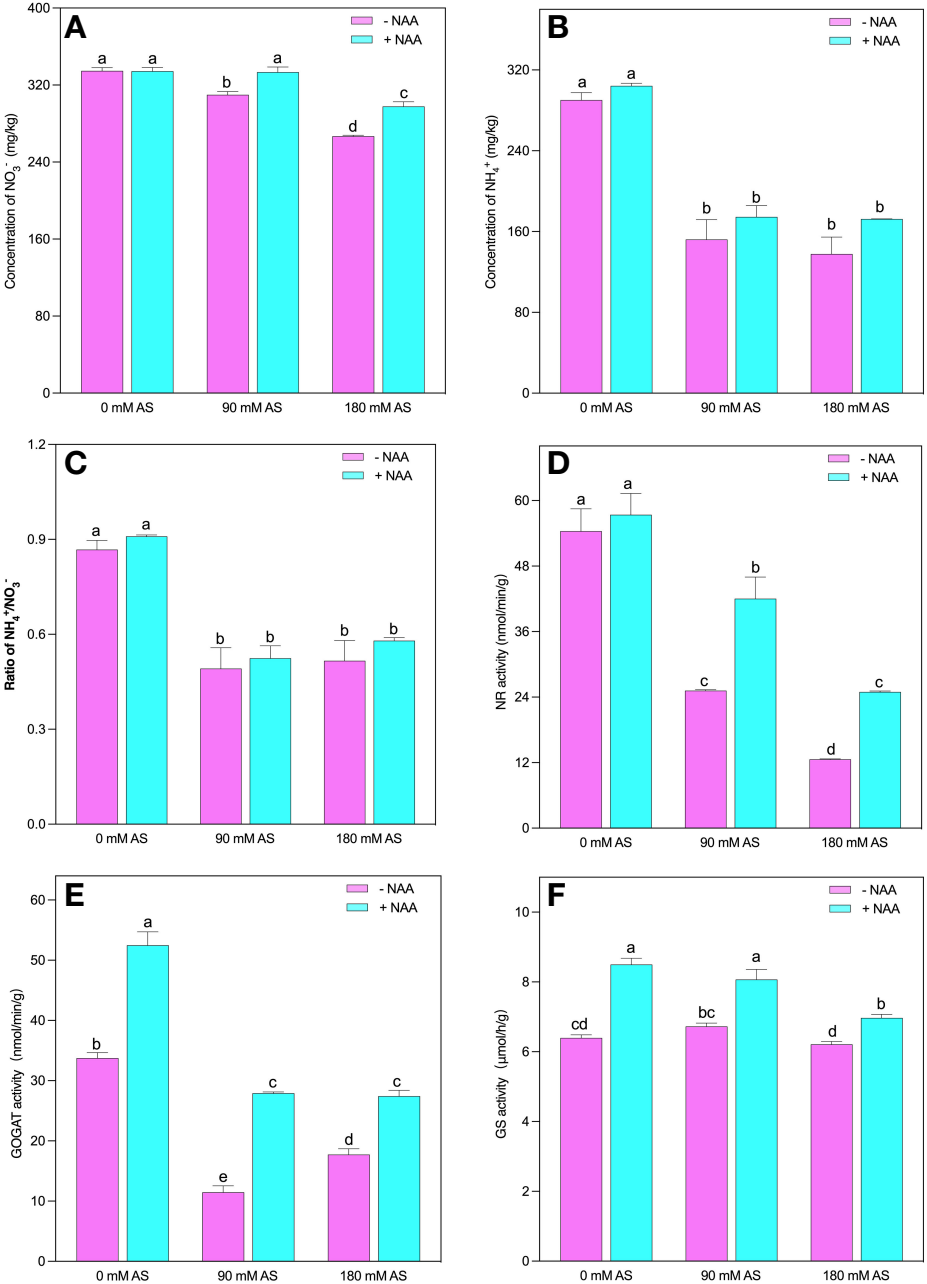
Changes in concentrations of **(A)** ${\text{NO}}_{3}^{-}$ and **(B)** ${\text{NH}}_{4}^{+}$ **(C)** ${\text{NH}}_{4}^{+}/{\text{NO}}_{3}^{-}$ ratio, and enzymatic activity of **(D)** NR **(E)** GS, **(F)** GOGAT in chufa plants under alkaline stress (AS) and exogenous naphthaleneacetic acid (NAA) application. Bars represents SD of mean, n=3. Different letters indicate significantly different values at *P*<0.05 (Duncan’s method).

The ${\text{NO}}_{3}^{-}$ concentration was not significantly influenced by the alkaline stress. Nonetheless, ${\text{NH}}_{4}^{+}$ and ${\text{NH}}_{4}^{+}/{\text{NO}}_{3}^{-}$ reduced substantially after alkaline stress. The 90 mM and 180 mM alkaline stress caused 47.5 and 52.5% reduction in ${\text{NH}}_{4}^{+}$ concentration, while, ${\text{NH}}_{4}^{+}/{\text{NO}}_{3}^{-}$ exhibited 43.3 and 40.5% inhibition, respectively (**Figures 5A–C**). Interestingly, no significant improvement was seen in ${\text{NO}}_{3}^{-}$ concentration under 0 mM alkaline stress; however, the 7.5 and 11.5% elevation was exhibited under respective alkaline levels. In addition, NAA application produced 5, 15 and 25% improvement in ${\text{NH}}_{4}^{+}$ and 5, 6 and 12% in ${\text{NH}}_{4}^{+}/{\text{NO}}_{3}^{-}$ under 0 mM, 90 mM and 180 mM alkaline stress, respectively. Moreover ${\text{NO}}_{3}^{-}$ reduction and ${\text{NH}}_{4}^{+}$ assimilation also decreased under alkalinity stress (**Figures 5D–F**).

The NR and GOGAT activities were inhibited by both alkaline levels. A 53.7 and 78% decline were observed in NR activities when the plants were exposed to 90 mM and 180 mM alkaline stress. This reduction was 66 and 48% for GOGAT activities in the respective alkaline levels. Additionally, the chufa. plants displayed 1.1-, 1.7- and 2-fold up-regulation in NR and 1.6-, 2.4- and 1.6-fold in GOGAT activities, under NAA foliar application, relative to untreated 0 mM, 90 mM and 180 mM alkaline stress, respectively (**Figures 5D, E**).

Although the alkaline stress could not induce any significant change in GS activities, the NAA application improved its activity by 1.3-, 12- and 1.2-fold under 0 mM, 90 mM, and 180 mM alkaline stress, respectively (**Figure 5F**).

### Changes in the osmoprotectants of chufa under alkaline stress followed by NAA application

**Figure 6:**
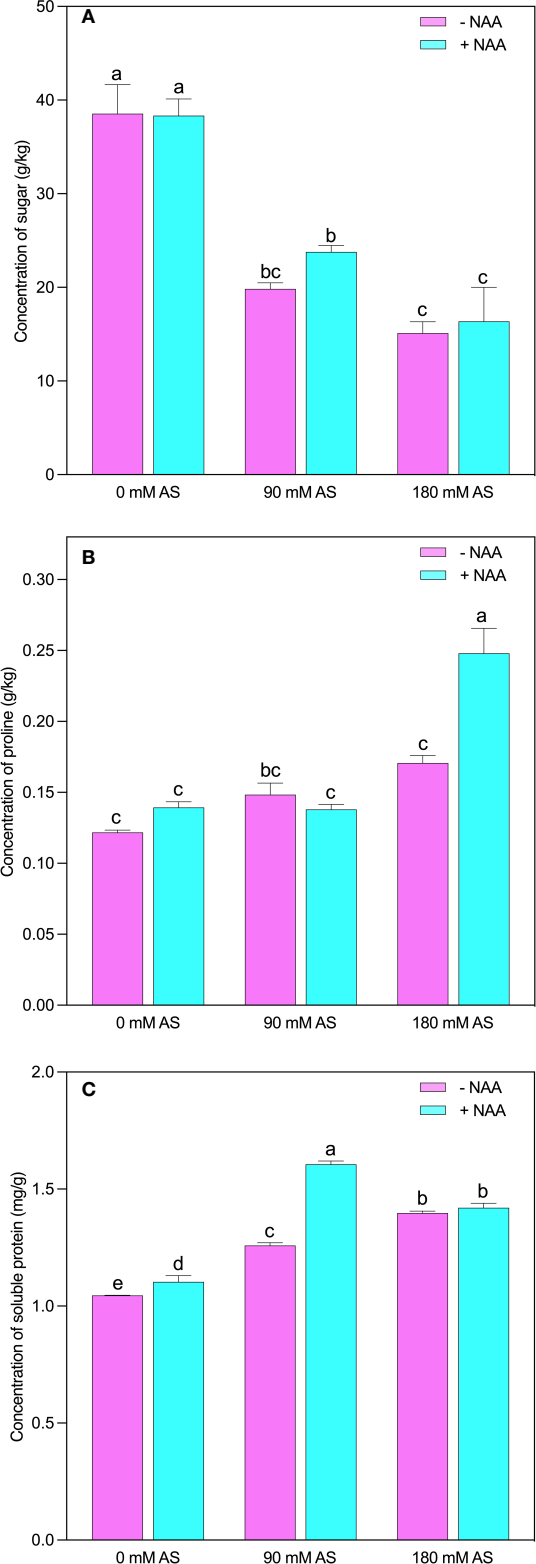
Changes in concentrations of **(A)** sugar, **(B)** proline and **(C)** soluble protein in chufa plants under alkaline stress (AS) and exogenous naphthaleneacetic acid (NAA) application. Bars represents SD of mean, n=3. Different letters indicate significantly different values at *P*<0.05 (Duncan’s method).

Likewise, in other parameters, the studied osmolytes were also influenced by the alkaline stress followed by NAA application (**Figures 6A-C**). The respective alkaline levels significantly reduced sugar contents (48.5 and 61%). Interestingly, the NAA could not improve the sugar contents at 0 mM and 180 mM stress levels, while a 19.4% up-regulation was recorded in sugar contents under 90 mM alkaline stress (**Figure 6A**). However, in the case of proline, this elevation (45%) was only detected after a 180 mM alkaline level (**Figure 6B**). The significant elevation in soluble protein (20.4 and 33.6%) and proline (21.3 and 40%) was observed after respective alkaline levels, respectively. The only significant up-regulation (27%) in soluble protein was found in the case of a 90 mM alkaline stress level (**Figure 6C**).

### Relationship between the investigated parameters

**Figure 7:**
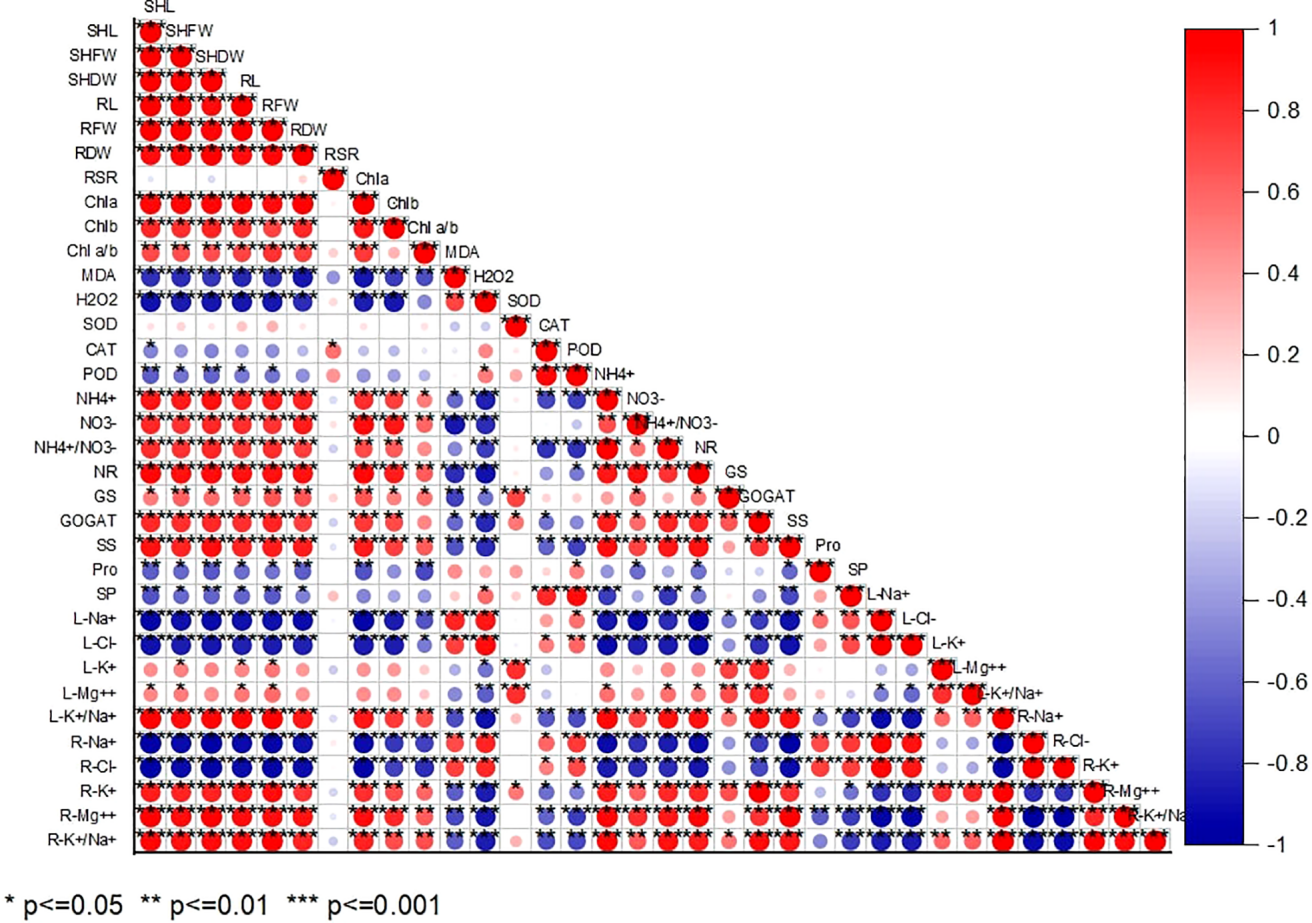
Pearson correlation analysis between study parameters in chufa plants under alkaline stress (AS) and exogenous naphthaleneacetic acid (NAA) application. SHL (Shoot lenght), SHFW (Shoot fresh shoot), SHDW (Shoot DW), RL (Root length), RFW (RFW), RDW (Root dry weight), Root-shoot ratio, Chla (Chlorophyll a), Chlb (Chlorophyll b), Chlab (Chlorophyll a/Chlorophyll b ratio), MDA (Malondialdehyde), H_2_O_2_, (Hydrogen peroxide), SOD (Superoxide dismutase), CAT (Catalase), POD (peroxidase), NR (Nitrate reductase), GS (Glutamine synthetase), GOGAT (Glutamine oxoglutarate aminotransferase), SS (soluble sugar), Pro (Proline), SP (Soluble protein), ${\text{NO}}_{3}^{-}$ (nitrate), ${\text{NH}}_{4}^{+}$ (ammonium), L-Na^+^ (Leaf N), L-Cl^-^ (Leaf Cl^-^), L-K^+^ (Leaf K^+^) L-Mg^++^ (Leaf Mg^++^), L-K^+^/Na^+^ ratio (Leaf K^+^/Na^+^ ratio), R-Na^+^ (Root N^+^), R-Cl^-^ (Root Cl^-^), R-K^+^ (Root K^+^) R-Mg^++^ (Root Mg^++^), R-K^+^/Na^+^ ratio (Root K^+^/Na^+^ ratio).

According to Pearson’s correlation analysis, all the studied growth indices positively correlated with Chl a, Chl b, Chla/b, NR, GS, GOGAT, ${\text{NH}}_{4}^{+}$ and ${\text{NO}}_{3}^{-}$ , ${\text{NH}}_{4}^{+}/{\text{NO}}_{3}^{-}$ , L-K^+^/Na^+^, R-K^+^, R-Mg^2+^, and R L-K^+^/Na^+^. While their negative correlation with MDA, H_2_O_2_, CAT, POD, Pro, SP, L-Na^+^, L-Cl^-^, R-Na^+^, and R-Cl^-^ indicates alkaline-induced oxidative stress persistence in chufa plant. (**Figure 7**).

## Discussion

Soil alkalinity (high pH) is an important abiotic stress, causing osmotic stress, ionic injuries, high pH-induced damages, and nutritional deficiencies, which result in acute physiological changes, thereby significantly affecting plant growth and productivity. Phytohormones have multiple functions in improving plant physiology both in normal growth conditions and in stress conditions (Fahad et al., 2015). The present study examined the effects of exogenously applied NAA on growth and physio-biochemical characteristics of chufa plants grown under different levels of simulated alkalinity stress.

We observed that alkaline stress significantly affected the growth and metabolism of chufa plants. For instance, the root and shoot length, root and shoot fresh weight, and dry weight were reduced significantly with increasing alkaline stress levels. The harmful effects of alkaline salt stress on growth parameters of chufa could be attributed to rise in pH, decrease in cell division and elongation, metabolic disruptions, nutrients deficiencies, and ionic imbalances which can be seen in **Figure 7** in the current experiment (Yang et al, 2009; Zhang and Mu, 2009; Abd-Alla et al., 2014; Mohsenian and Roosta, 2015; Li et al., 2019; Ullah et al., 2019). However, root-shoot ratio (RSR) non-significantly increased compared to controlled conditions. Increased RSA may be an adaptive response that provides a greater capacity for water and nutrient absorption (Lv et al., 2013). However, exogenous NAA application improved growth parameters of alkaline-stressed chufa plants, demonstrating that NAA’s ameliorative action in reducing alkaline stress damages. NAA, similar to naturally occurring IAA stimulates cell division, and cell elongation leading to increased growth. Consequently, the results of the current study and previously published reports indicate that NAA provides protection against a wide variety of environmental stresses (Zhou et al., 2012; Abou El-ghit, 2015; Ullah & Sajjad, 2017; Muhammed et al., 2022).

The pigment chlorophyll is essential for photosynthesis. Plant growth is most commonly reduced under alkalinity conditions due to a decrease in photosynthetic capacity (Ullah et al., 2019). In the present study, we observed that alkaline stress significantly decreased chlorophyll concentrations (Chl a and Chl b) which might be the result of (*a*) Mg^2+^ precipitation that degrades green pigments, (*b*) increased oxidative stress causing damage to chloroplasts, and (*c*) increased chlorophyllase activity, which is responsible for chlorophyll destruction (Kariola et al., 2005; Abdel Latef and Chaoxing, 2011; Fu et al., 2018). Compared to unsprayed chufa plants, NAA increased chlorophyll concentrations under controlled and alkaline stress conditions. Several studies have shown that NAA reduces the damage caused to chlorophyll pigments in leaves by saline-alkaline stress, which is in agreement with our findings (Burondkar et al., 2009; Abou El-ghit, 2015; Ullah and Sajjad, 2017; Muhammed et al., 2022). Importantly, NAA-induced restoration of chlorophyll pigments in the alkaline-stressed chufa plants exhibited an osmoprotective and membrane-protective role of NAA for chufa plants subjected to alkalinity. Our findings suggest that, the increase in chlorophyll pigments caused by NAA could be the result of its ability to promote pigment synthesis and retard pigment degradation by increasing antioxidant capacity or by stimulating the synthesis of stabilizing substances.

In the current study, an increase in alkaline stress caused an increase in Na^+^ and Cl^-^ concentration while a decrease in Mg^2+^ and K^+^ concentration, low K^+^/Na^+^ ratio in both roots and leaves as shown by the **Figure 7**. There is evidence that alkalinity causes high pH in the rhizosphere which reduces the availability of ions of nutrient elements in the soil, such as K^+^, Mg^2+^, Ca^2+^, and ${\text{H}}_{2}{\text{PO}}_{4}^{-}$ by causing their precipitation (Yang et al., 2007). The alkalinity-induced reduction in K^+^ and Mg^2+^ concentration is likely due to stress-driven repression of K^+^ and Mg^2+^ absorption. Further, the reduced K^+^ and K^+^/Na^+^ ratio could also be attributed to the competition between K^+^ and Na^+^ ions for binding sites necessary for cellular functions (Azooz et al., 2015; Ullah et al., 2019). Toxic accumulations of salt ions compromise plant development, metabolism and growth. We suggest that, the poor growth of chufa plants as a consequence of alkaline stress might be the result of osmotic, ionic, and pH-induced cellular membrane damage, associated with higher levels of Na^+^ and Cl^-^ ions and a reduction in beneficial K^+^ and Mg^2+^ ions (Zhu, 2001; Chartzoulakis 2005; Ullah et al., 2019; Noor et al., 2022). During transpiration, the fast-moving xylem carries Na^+^ and Cl^-^ to the shoots (Tester and Davenport, 2003). Consequently, we suggest that chufa leaves accumulated more Na^+^ and Cl^-^ ions than roots, making them more susceptible to salt ions. The results of our study agree with those of previous studies, which reported significantly lower levels of Na^+^ and K^+^ in roots compared to shoots (Wang et al., 2012; Menezes et al., 2017). Our work suggests that chufa plants use these excess accumulations of Na^+^, Cl^-^, K^+^, and Mg^2+^ to survive under water deficit conditions. For example, these excess ions might act as osmolytes for reducing leaf water to enhance water absorption and improve their photosynthesis and other metabolic processes.

In contrast, NAA-applied chufa plants significantly decreased Na^+^ and Cl^-^ concentrations while improving Mg^2+^, K^+^, and K^+^/Na^+^ ratios in both roots and leaves. Previous studies reported that IAA and NAA reduced the Na^+^ accumulation and enhanced *K* ^+^, Ca^+2^, and P contents in plants grown under salinity conditions (Kaya et al., 2010; Olaiya and Anyanwu, 2013; Muhammed et al., 2022). Therefore, we propose that NAA application ameliorated the adversity of alkalinity on *C. esculentus* by declining the toxic accumulations of salt ions and increasing beneficial mineral nutrients (i.e., K^+^, Mg^2+^) to optimize its cellular metabolism and growth. Considering the high K^+^/Na^+^ ratio, it appears that exogenous NAA application has a balancing effect on K^+^ and Na^+^ uptake under alkaline stress, suggesting that NAA may be used as an alleviating agent in alkaline soil grown crops.

Alkalinity stress often causes plants to experience oxidative stress damages and membrane lipid peroxidation (Gong et al., 2013; Guo et al., 2019). In our study, alkaline stress increased concentration of H_2_O_2,_ resulted in higher MDA accumulation, which strongly indicates ROS bursts and potential oxidative damage of chufa plant cells, indicating that there is a positive correlation between MDA, H_2_O_2_ and other oxidative stress biomarkers as indicated in **Figure 7** (Ahmad et al., 2014; Guo et al., 2019). However, alkalinity-stressed chufa plants significantly upregulated the activity of SOD, POD and CAT. There is evidence that plants upregulate several antioxidant enzymes (SOD, POD and CAT) which contribute significantly to the metabolism of excessive ROS under alkalinity stress (Zhang and Mu, 2009; Gong et al., 2013; Fu et al., 2017; Guo et al., 2019), which corroborates our findings. In contrast, NAA application significantly inhibited the accumulation of H_2_O_2_ and MDA, but increased activity of antioxidant enzymes including SOD, CAT and POD in alkalinity-stressed chufa plants compared with control plants and alkalinity-treated plants alone, suggesting that NAA activated adaptive mechanisms against oxidative damages in stressed plants. These findings demonstrate that NAA-application increased antioxidant activity to protect chufa plants against oxidative damage associated with alkalinity, as evidenced by the observed decrease in MDA concentration, which are in line with previous studies (Olaiya & Anyanwu, 2013; Ullah & Sajjad, 2017; Khedr et al., 2022; Muhammed et al., 2022). We suggest that chufa plant is able to significantly increase the activity of antioxidant enzymes in order to resist alkaline stress. However, this, diverting energy and protein into anti-oxidant mechanisms come at a significant cost in terms of growth.

In addition to oxidative stress, alkaline stress also causes osmotic stress in sensitive plants. In response, plants accumulate compatible solutes, such as glycine betaine, proline, and soluble sugars, to regulate osmotic pressure and enhance stress tolerance (Yang et al., 2007; Wang et al., 2012; Guo et al., 2017). In our study, chufa plants were significantly enriched in soluble protein and proline under alkaline stress, and NAA application further increased their concentration. Hence, it seems that the accumulation of soluble protein and proline as osmolytes played a critical role in the physiological response of chufa plants to alkaline stress. In addition, exogenous NAA application to alkalinity-stressed chufa plants might have further facilitated the stabilization of membranes, enzymes, and proteins, and elimination of excess ROS for protecting the photosynthetic machinery (Verdoy et al., 2006; Ahmad et al., 2016; Muhammed et al., 2022). Furthermore, soluble sugar levels decreased as alkalinity stress increased; however, NAA application increased the concentration of soluble sugar compared to unsprayed alkalinity-stressed chufa plants. There have been reports that exogenous application of NAA and IAA increases the proline and soluble sugar under salinity stress for various plant species, which is consistent with our observation (Ullah & Sajjad, 2017; Mir et al., 2020). Soluble sugars and proline accumulate during stress, contributing to the maintenance of metabolism, for example, by alleviating ROS-induced oxidative stress damages (Sami et al., 2016). Moreover, in alkalinity-subjected chufa plants, high levels of soluble protein may serve as a form of nitrogen storage that can be reclaimed when stress is relieved (Abdel Latef, 2010; Abdel Latef and Chaoxing, 2014; Zhang et al., 2014). The increase in soluble protein was accompanied by a significant decline in growth of plants subjected to alkalinity stress. Therefore, our results suggest that chufa plants utilize most of the synthesized proteins for osmoregulation rather than growth in response to alkaline stress.

Plants typically absorb nitrogen (N) derived from inorganic sources, such as ${\text{NO}}_{3}^{-}$ and ${\text{NH}}_{4}^{+}$ ${\text{NH}}_{4}^{+}$ , through their roots which are then distributed for cellular functions. We observed that both ${\text{NO}}_{3}^{-}$ and ${\text{NH}}_{4}^{+}$ levels decreased with increasing alkalinity stress gradients as presented in **Figure 7**. A plethora of studies demonstrated that alkalinity inhibits ${\text{NO}}_{3}^{-}$ and ${\text{NH}}_{4}^{+}$ uptake, resulting in low concentrations (Yang et al., 2008; Yang et al., 2009; Yang et al., 2010; Ullah et al., 2019). Several transport systems utilize by roots of plants to absorb ${\text{NO}}_{3}^{-}$ and ${\text{NH}}_{4}^{+}$ For instance, ${\text{H}}^{+}{\text{/NO}}_{3}^{-}$ symport mediates ${\text{NO}}_{3}^{-}$ uptake through a transmembrane proton gradient (Crawford and Glass, 1998). The alkalinity-induced decrease in ${\text{NO}}_{3}^{-}$ uptake by roots might be the result of high pH injuries, resulting in large reduction of OsNR1 expression in roots of rice seedlings (Wang et al., 2012). Further, the AMT protein regulates the absorption of ${\text{NH}}_{4}^{+}$ (Crawford and Glass, 1998; Wang et al., 2012). Further, it has been reported that alkalinity (pH, 9.11) reduces ${\text{NH}}_{4}^{+}$ for root uptake in the surrounding rhizosphere by changing it to NH_3_ , or decreases ${\text{NO}}_{3}^{-}$ concentrations and OsNR1expression, resulting in a reduction in ${\text{NH}}_{4}^{+}$ synthesis (Wang et al, 2012). Hence, we suggest that alkaline salt stress might hamper the activity of NRT and AMT, resulting in a decreased uptake of ${\text{NO}}_{3}^{-}$ and ${\text{NH}}_{4}^{+}$ . There is a possibility that this phenomenon may affect virtually all processes of plant metabolism (Guo et al., 2017).

The ${\text{NO}}_{3}^{-}$ is further reduces into nitrite $\left(NO_{2}^{-}\right)$ and then to ${\text{NH}}_{4}^{+}$ by the actions of nitrate reductase (NR) and nitrite reductase respectively. Further incorporation of ${\text{NH}}_{4}^{+}$ into organic molecules is carried out by glutamine synthetase and glutamate synthase cycle (GS/GOGAT) or alternatively *via* glutamate dehydrogenase (GDH) (Shi et al., 2009). The present study indicated significant decreases in NR and GOGAT activities due to increased alkaline stress, while there was no significant effect on GS activity. Alkalinity stress has been reported to interfere in ${\text{NO}}_{3}^{-}$ reduction and ${\text{NH}}_{4}^{+}$ assimilation by affecting the activities of NR, NiR, GS/GOGAT and GDH enzymes in plants (Song et al., 2006; Yang et al., 2007; Wang et al., 2012; Zhang et al., 2013; Guo et al., 2017; Ullah et al., 2019), which corroborates our findings. The decrease in in ${\text{NO}}_{3}^{-}$ and ${\text{NH}}_{4}^{+}$ assimilation hampers N metabolism and amino acid synthesis, leading to reduced plant growth and dry weight (Queiroz et al., 2012; Ullah et al., 2019). The observed decline in NR activity could be attributed to decreased carbon fixation, decreased ${\text{NO}}_{3}^{-}$ uptake by roots, or its low translocation in the xylem, which consequently decreases ${\text{NO}}_{3}^{-}$ availability to plants (Kumar and Joshi, 2008; Gloser et al., 2020). In addition, there may be a negative effect of alkalinity stress on the enzyme protein synthesis and/or activity that contributes to the decrease in NR activity. Further, the decrease in GOGAT activity at both stress levels and GS at high stress level, could be attributed to an increase in protein oxidation (Balestrasse et al., 2006). Due to the fact that alkalinity stress is known to stimulate the generation of ROS which may contribute to the destruction of these enzyme proteins by oxidative stress (Zhang and Mu, 2009). In contrast, NAA-applied chufa plants increased the concentration of ${\text{NO}}_{3}^{-}$ and ${\text{NH}}_{4}^{+}$ as well as up-regulated NR, GS and GOGAT enzymes in leaves of chufa plants. Additionally, Sharma and Dubey (2005) demonstrated that NR is highly sensitive to H_2_O_2_. Therefore, we suggest that, NAA application indirectly increased NR enzymes by reducing the accumulation of excessive H_2_O_2_ under alkalinity stress condition. In addition, NAA-induced increase in GS and GOGAT activities in in leaves of chufa plants subjected to alkalinity stress, indicating its role in proper incorporation of ${\text{NH}}_{4}^{+}$ into glutamate pool and further amino acid biosynthesis.

In plants, nitrogen is an integral component of nucleic acid, amino acids, proteins, and chlorophyll pigments and secondary metabolites. Hence, it plays a significant role in numerous physio-biochemical mechanisms. In fact, the regulation of N metabolism contributes to stress tolerance through various mechanisms (Rais et al., 2013; Arghavani et al., 2017). We therefore propose that NAA application facilitated the N assimilation of alkalinity stressed chufa plants, by increasing the concentration of ${\text{NO}}_{3}^{-}$ and ${\text{NH}}_{4}^{+}$ as well as their metabolizing enzymes, thus improving their tolerance to alkalinity stress. Further, we noticed that NAA application increased N assimilation along with enzymatic antioxidants in alkalinity-stressed chufa plants. Consequently, we propose that alkalinity stressed chufa plants need amelioration of N assimilation for maximizing protein synthesis needed to divert to anti-oxidant mechanism for protecting the cells from alkalinity induced oxidative stress damage. Consequently, we propose that chufa NAA-application to alkalinity stressed chufa plants ameliorated their N assimilation, enabling them to synthesize the maximum amount of protein necessary to enhance their anti-oxidant mechanism against alkalinity-induced oxidative stress. The increased N assimilation, protein concentration, and increased enzymatic anti-oxidant mechanisms, and reduced H_2_O_2_ and MDA concentration in NAA-applied alkalinity stressed chufa plants could explain this conclusion.

## Conclusion

We used exogenous NAA application to check whether it can alleviate the adverse effects of alkalinity on the growth and physio-biochemical features of chufa plants. We found that exogenous application of NAA promoted the growth and metabolism of chufa by reducing the damage of alkalinity stress. For instance, exogenous NAA resulted in (i) reduction of toxic salt ions (Na^+^ and Cl^-^), (ii) protection of photosynthetic apparatus (iii), maintenance of beneficial mineral ions (K^+^ and Mg^2+^), (iv) reduction of ROS and lipid peroxidation by upregulating enzymatic antioxidant defense mechanism (v) improvement of nitrogen assimilation, and (vi) stimulation of osmolytes. In addition, chufa leaves accumulated a larger quantity of Na^+^, Cl^-^, K^+^, and Mg^2+^ ions than roots, suggesting that leaves are capable of increasing their cellular osmolality, thereby facilitating the upward flux of water from the soil to leaves; an important strategy for adaptation to hyper-arid conditions. Our results show that NAA acts as a powerful plant growth regulator capable of modulating chufa growth and physiological responses. We suggest that application of exogenous NAA may therefore be a an effective strategy for reducing alkalinity-induced negative effects on chufa and other salt-sensitive vital economic crop species in the hyperarid Taklamakan desert, where salty groundwater is the sole source of agricultural irrigation water.

## Data availability statement

The original contributions presented in the study are included in the article/**Supplementary Material**. Further inquiries can be directed to the corresponding authors.

## Author contributions

Conceptualization: AU, FZ. Data curation: AU. Formal analysis: AU, MA, and JN. Funding acquisition; FZ. Investigation: AU, AT. Methodology: AU. Project administration; FZ. Resources: FZ. Software: AU, AT, JN, MAA, KS, AR, and ZZ. Supervision: FZ. Validation: FZ, AU, AT, and ZZ. Visualization: JN, KS, AR, MN, and MA. Writing - original draft: AU. Writing - review and editing: AU, FZ, AT, ZZ, JN, MN. All authors contributed to the article and approved the submitted version.

## Funding

National Key Research and Development Project of China (No. 2019YFC0507603), and Natural Science Foundation of Xinjiang Uygur Autonomous Region (No.2021D01D02), jointly supported this work.

## Conflict of interest

The authors declare that the research was conducted in the absence of any commercial or financial relationships that could be construed as a potential conflict of interest.

## Publisher’s note

All claims expressed in this article are solely those of the authors and do not necessarily represent those of their affiliated organizations, or those of the publisher, the editors and the reviewers. Any product that may be evaluated in this article, or claim that may be made by its manufacturer, is not guaranteed or endorsed by the publisher.
