# Supplementary material for: Exogenous naphthaleneacetic acid alleviated alkalinity-induced morpho-physio-biochemical damages in Cyperus esculentus L. var. sativus Boeck
Source: Front Plant Sci. 2022 Oct 18;13:1018787. doi: 10.3389/fpls.2022.1018787 (PMC9624244; doi:10.3389/fpls.2022.1018787)
Supplement: Supplementary file 1 [file Table_1.docx]

A preliminary growth experiment was conducted with NAA concentrations ranging from 50 to 150 mg/L. An optimal concentration of NAA was selected based on improved growth of plants in a solution containing 90 mM of alkaline stress.

Effect of different NAA concentration on Chufa seedling under induced-alkalinity stress for 96 h.

| Treatments | Plumule length (cm) | Plumule fresh weight (g) | Plumule dry weight (g) | Radicle length (cm) | Radicle fresh weight (g) | Radicle dry weight (g) |
| --- | --- | --- | --- | --- | --- | --- |
| 0 mM alkalinity | 8.20 ± 0.17a | 0.24 ± 0.01a | 0.07 ± 0.00a | 5.37 ± 0.12a | 0.20 ± 0.01a | 0.04 ± 0.00a |
| 90 mM alkalinity | 4.03 ± 0.18e | 0.17 ± 0.01c | 0.02 ± 0.00c | 2.90 ± 0.35c | 0.14 ± 0.00b | 0.02 ± 0.00c |
| 90 mM alkalinity + 50mg NAA | 4.87 ± 0.15d | 0.18 ± 0.01bc | 0.03 ± 0.00c | 3.27 ± 0.09c | 0.15 ± 0.01b | 0.02 ± 0.00c |
| 90 mM alkalinity + 100mg NAA | 5.40 ± 0.12c | 0.18 ± 0.00bc | 0.04 ± 0.00b | 3.37 ± 0.09c | 0.15 ± 0.01b | 0.03 ± 0.00b |
| 90 mM alkalinity + 150mg NAA | 6.00 ± 0.15b | 0.20 ± 0.01b | 0.05 ± 0.00b | 4.07 ± 0.09b | 0.16 ± 0.01b | 0.03 ± 0.00b |

Values are mean ± SE. Different letters indicate significant differences at *P* < 0.05 based on Duncan multiple range test.
